# Supplementary material for: Propagation Control of Octahedral Tilt in SrRuO3 via Artificial Heterostructuring
Source: Adv Sci (Weinh). 2020 Jun 25;7(16):2001643. doi: 10.1002/advs.202001643 (PMC7435247; doi:10.1002/advs.202001643)
Supplement: Supplementary file 1 — Supporting Information [file ADVS-7-2001643-s001.pdf]

WILEY-VCH

Copyright WILEY-VCH Verlag GmbH & Co. KGaA, 69469 Weinheim, Germany, 2020.

Supporting Information

Propagation control of octahedral tilt in $SrRuO_3$ via artificial heterostructuring

*Seung Gyo Jeong, Gyeongtak Han, Sehwan Song, Taewon Min, Ahmed Yousef Mohamed, Sungkyun Park, Jaekwang Lee, Hu Young Jeong, Young-Min Kim, Deok-Yong Cho, and Woo Seok Choi**

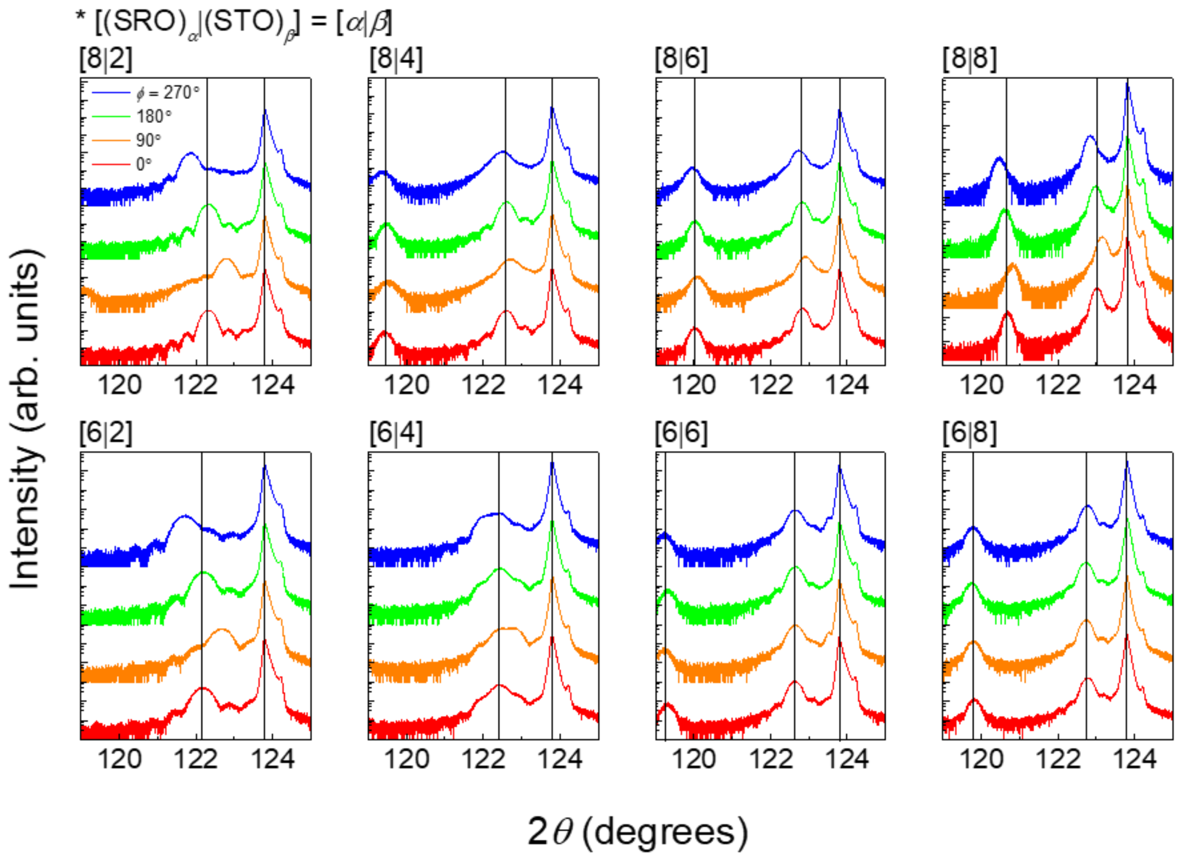

Figure S1. Structural characterization of the [α|β] SLs. Off-axis X-ray diffraction measurements for the [α|β] SLs around the STO (204) Bragg reflections with φ angles of 0, 90, 180, and 270°. The vertical lines are guides to the eye.

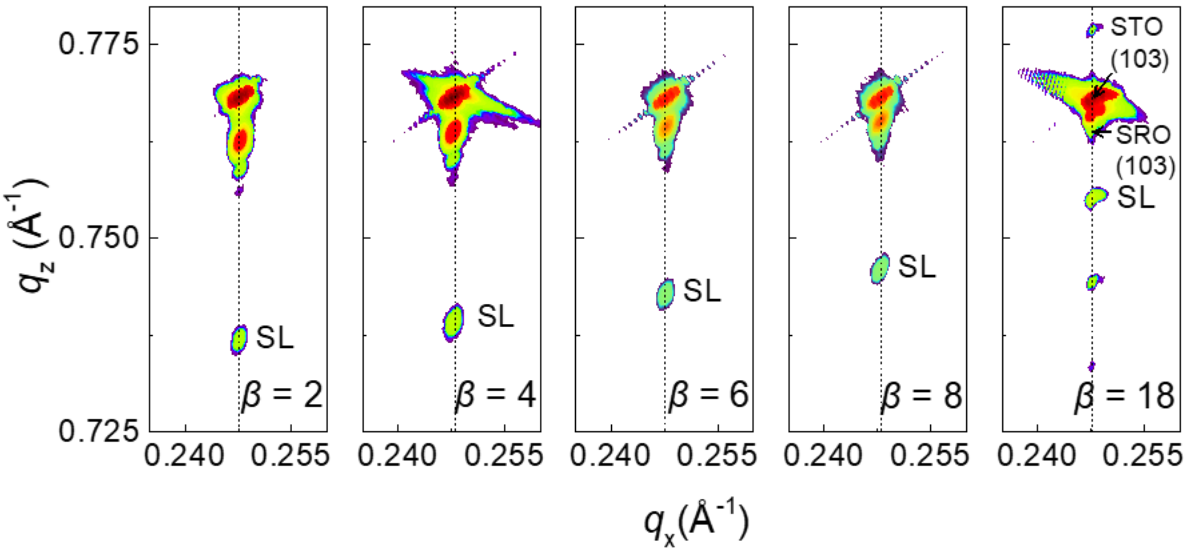

Figure S2. Epitaxial strain of [6|*β*] SLs. XRD RSMs of the SLs, shown for the [6|*β*] SL around the (103) Bragg reflection of the STO substrate, indicating the fully strained state of the SLs with the coherent in-plane lattice constant as that of the substrates.

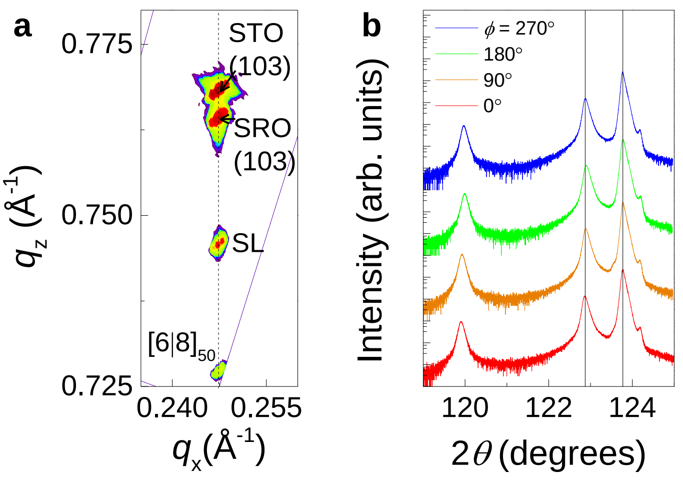

Figure S3. XRD results of $[6|8]_{50}$ SL, 6 u.c. layers of SRO and 8 u.c. layers of STO repeated 50 times along the growth direction, are shown. a) RSM and b) off-axis measurements consistently indicate that the tetragonal SRO is well maintained up to ~120 nm of SRO thickness with a fully strained state.

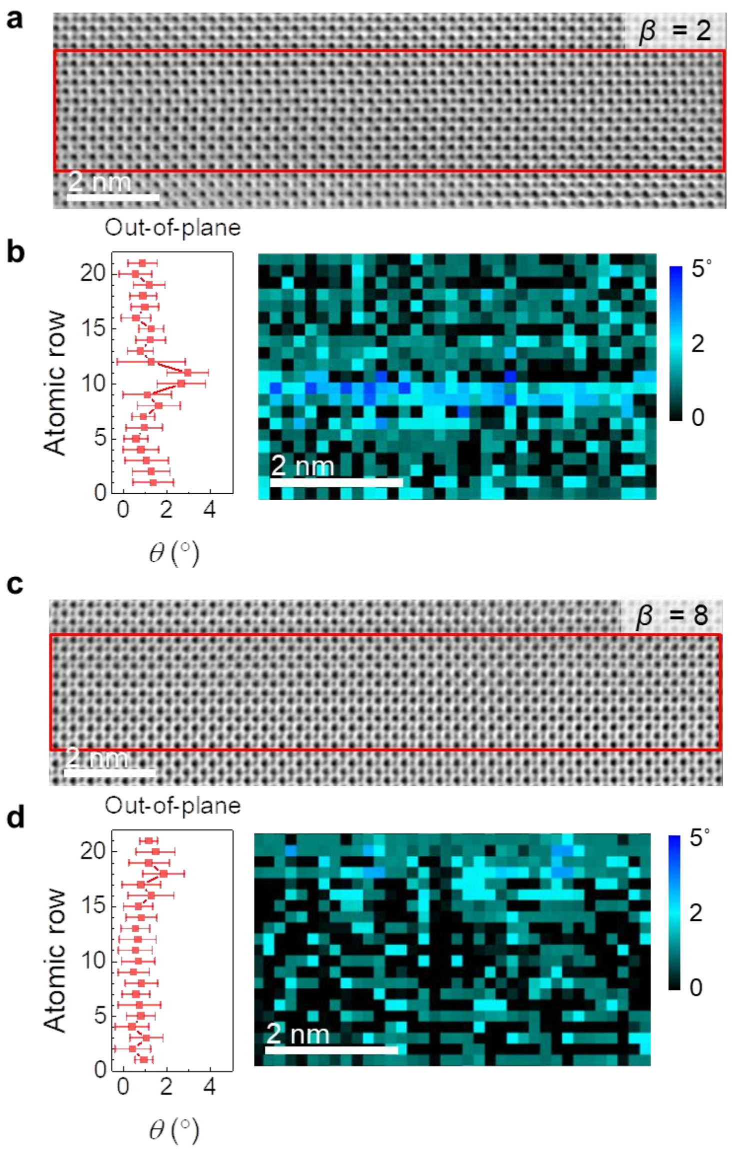

Figure S4. ABF-STEM images of [6|*β*] SLs in high magnification. ABF-STEM observation also displays the well-defined epitaxy of the SLs with a) $\beta = 2$, and c) 8, with a clear visualization of the oxygen atoms, respectively. We extracted the average *M*-O bonding angles (θ) (left panel) of the SLs with b) $\beta = 2$, and d) 8, along the out-of-plane direction from the contour plot (right panel), respectively.

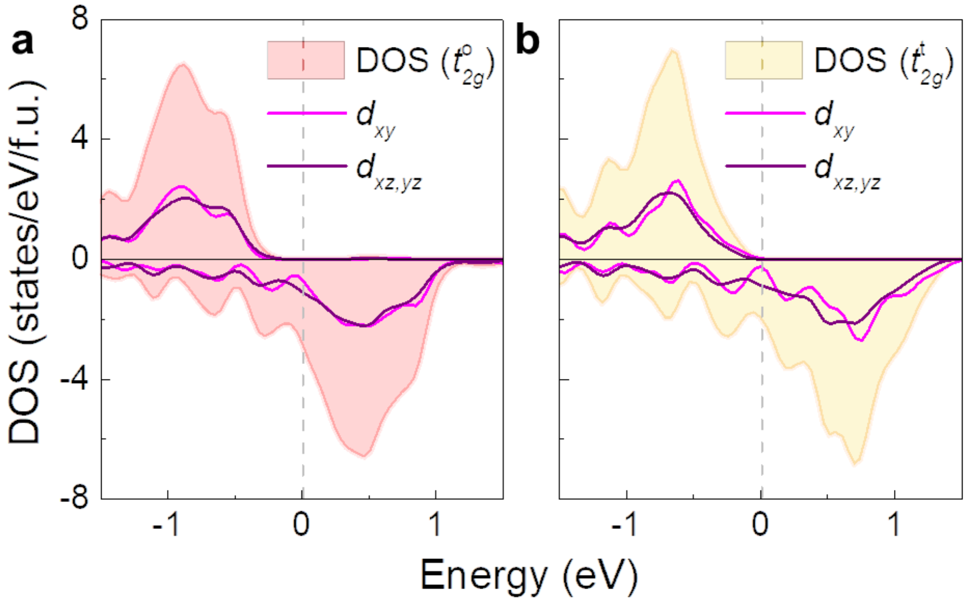

Figure S5. Orbital selective PDOS of Ru-t_{2g} states for a) orthorhombic (DOS (t_{2g}^{o})) and b) tetragonal SRO (DOS (t_{2g}^{t})). The vertical dashed lines indicate the Fermi level.

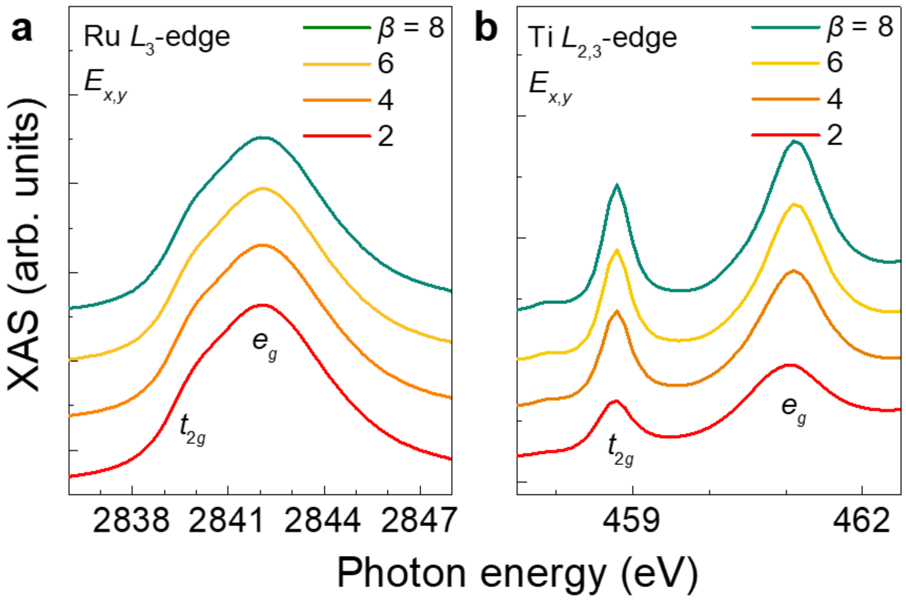

Figure S6. XAS spectra for the [6|β] SLs. a) Ru L_3-edge XAS spectra of [6|β] SLs with different β values are acquired using a normal incident beam into the film surface ($E_{x,y}$), in which the electrical field of X-rays lies along the vertical direction in the measurement chamber. The t_{2g} and e_g energy levels of the Ru L_3-edge are assigned at ~2839.5 eV and ~2842 eV, respectively. b) The t_{2g} and e_g energy levels of the Ti $L_{2,3}$-edge are assigned at ~458.8 eV and ~461.1 eV, respectively.
